# The role of molecular profiling for castration-resistant prostate cancer treatment and therapy development

**DOI:** 10.3389/fcell.2026.1894354

**Published:** 2026-08-21

**Authors:** Timothy J. Mann, Therese M. Becker, Tara L. Roberts, Paul de Souza, John G. Lock

**Affiliations:** 1 School of Biomedical Sciences, University of New South Wales, Sydney, NSW, Australia; 2 Ingham Institute for Applied Medical Research, Liverpool, NSW, Australia; 3 School of Medicine, Western Sydney University, Penrith, NSW, Australia; 4 South Western Sydney Clinical School, University of New South Wales, Liverpool, NSW, Australia; 5 Nepean Clinical School, University of Sydney, Sydney, NSW, Australia

**Keywords:** castration-resistant prostate cancer, liquid biopsy, molecular profiling, precision oncology, tumor hetereogeneity

## Abstract

Early-stage prostate cancer (PCa) is overwhelmingly driven by aberrant androgen receptor (AR) signalling. Accordingly, standard-of-care treatments include androgen deprivation therapy (ADT) and AR pathway inhibitors (ARPIs), with disease detection and therapy-response monitoring relying on blood-borne PSA, a product of AR transcriptional programs. Crucially, resistance to these therapies is inevitable but heterogeneous in nature; potentially driven by a diverse array of alternate signalling pathways and differentiation mechanisms such as neuroendocrine conversion. Here, we review the current standard of care for CRPC and highlight this heterogeneity - between and even within patients - which demands a new paradigm to longitudinally monitor the evolving molecular profiles of each patient to guide rational selection of targeted therapies, as well as rational patient stratification to optimise clinical trials for emerging therapies. Since solid biopsies are incompatible with extensive longitudinal profiling, we here consider recent advances in liquid biopsy-based profiling methods and assess their potential as cornerstones in a new paradigm of personalised, adaptable disease monitoring and therapeutic decision-support.

## Introduction

**FIGURE 1:**
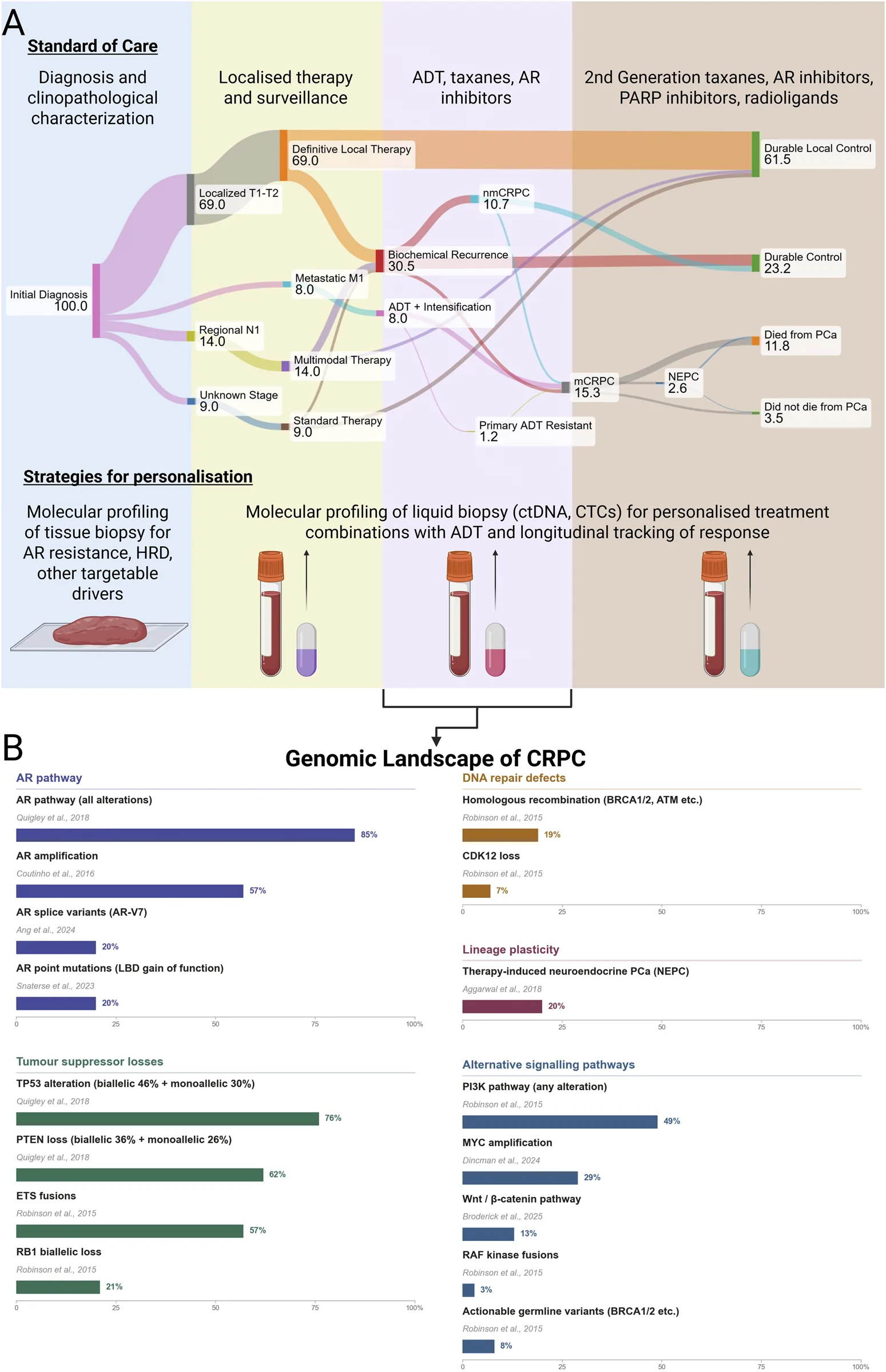
**(A)** Disease progression of PCa patients from diagnosis, expressed as a percentage. Headings describe standard of care, below describes potential strategies for personalised medicine. Competing non-PCa mortality not shown. Extrapolated from US and European studies (SEER 2021, Kolodziej 2014, Hayman et al., 2019, Shore et al., 2024, Sweeney et al., 2015, Conteduca et al., 2019, Varenhorst et al., 2016, Aggarwal et al., 2018, Siegel et al., 2024). **(B)** Genomic alterations associated with development of CRPC. Grouped as AR pathway alterations, tumor suppressor losses, DNA damage repair defects, lineage plasticity and alternative signalling pathways. Bars indicate the approximate prevalence of each alteration in CRPC tumours, compiled from multiple cohorts and methodologies (Quigley et al., 2018; Coutinho et al., 2016; Snaterse et al., 2023; Robinson et al., 2015; Aggarwal et al., 2018; Dincman et al., 2024; Broderick et al., 2025; Ang et al., 2024, Guo et al., 2022). Established therapies are noted when applicable.

In men, prostate cancer (PCa) is the second most commonly diagnosed and fifth most lethal cancer, with an estimated 1.47 million new cases and almost 397,000 deaths globally in 2022 (Bray et al., 2024). Early-stage PCa has favourable prognosis, with 5-year survival rate of 97.9% (Howlader et al., 2021), since surgery and radiation are effective treatments for localised disease. However, 20%–30% of patients relapse after 5 years following initial therapy (Kolodziej, 2014); with rising prostate specific antigen (PSA) heralding the onset of metastatic disease, the large majority of which is driven by AR signalling. Treatment resistance inevitably occurs after the initial use of leutinising hormone releasing hormone (LHRH) agonists; 10%–20% of patients progress to castration-resistant prostate cancer (CRPC) within 2 years of androgen deprivation therapy (ADT)-initiation (Hussain et al., 2024), with virtually all of these patients eventually progressing to CRPC and then ultimately to incurable metastatic CRPC (mCRPC). When reviewing options for the treatment of CRPC, it is important to first dissect the highly heterogenous patient journey that leads to this incurable disease, and potential strategies to mitigate this progression (Figure 1).

## Patient journey: the path to castration resistance

Initial detection of PCa is primarily through serum measurement of PSA, a canonical transcriptional target intrinsically linked to AR signalling (Tan et al., 2015). AR is a transcription factor that is activated upon binding testosterone or dihydrotestosterone (DHT), translocating to the nucleus to then activate transcription of genes that drive cell proliferation, and survival (Tan et al., 2015).

Collectively, PSA levels, tumor-node-metastasis (TNM) stage classification, imaging via prostate specific membrane antigen–positron emission tomography (PSMA-PET) and Gleason score (histological grading) are traditionally used to stratify patients into clinical risk groups that guide first line-management (Cornford et al., 2024).

## Localised and biochemically recurrent PCa

Low risk disease is commonly managed with active surveillance, while intermediate and high risk PCa is treated with definitive therapies such as radiotherapy or radical prostatectomy (RP). Definitive therapies may also be combined with androgen deprivation therapy (ADT) – which lowers circulating androgens by 90%–95% (Nishiyama, 2014), therefore reducing AR-driven transcription.

## Metastatic PCa

Patients with metastatic castration-sensitive PCa (CSPC) are typically administered systemic ADT in combination with second generation AR pathway inhibitors (ARPIs) such as enzalutamide, apalutamide or darolutamide, which function by competitively blocking androgen binding to AR and inhibiting nuclear translocation resulting in further suppression of AR over ADT-alone (Ong et al., 2021). ARPIs may be administered in combination with docetaxel, a taxane that binds β-Tubulin and arrests cell cycle leading to G2/M arrest and subsequent apoptosis (Pienta, 2001).

In a recent cohort of 16,311 patients that underwent definitive therapy (either RP or radiotherapy), 70%–85% achieved durable disease control at 10 years without biochemical recurrence (BCR), with no statistical differences between RP or radiotherapy when adjusted for risk group (Falagario et al., 2023). For those patients that develop BCR, salvage ADT is initiated. Approximately 85%–90% of treatment naïve metastatic PCa biochemically respond to ADT within weeks as measured by PSA decline to <0.2 ng/mL or a 50% reduction. However, the inevitable progression to CRPC occurs at a median of 18–24 months, (Karanika et al., 2015).

Upon progression to CRPC, further ARPIs or a switch in ARPIs is administered, with PSA response rates in 54% of ARPI naïve patients on enzalutamide (Scher et al., 2012) and 29% on abiraterone (de Bono et al., 2011). These response rates are dependent upon prior treatments and disease context, however development of mCRPC is inevitable, where patients are managed with targeted therapies alone or in combination with ADT or ARPIs.

## Early molecular testing and treatment

Each branchpoint in Figure 1 represents a therapeutic decision, highlighting the need for safe, longitudinal and comprehensive molecular profiling to guide optimal management. While staging and grading systems (TNM, Gleason) are used to stratify initial cancer aggressiveness and patient management, molecular profiling remains largely absent from routine clinical practice, presumably due to highly effective definitive therapies for most men. However, reliance on clinicopathological parameters leaves AR inhibition as the only therapeutic axis, effectively selecting tumour evolution towards incurable mCRPC, upon development of recurrent disease. It is likely that the most effective strategy for managing CRPC may be to limit, or at least delay, its development in the first place.

As radiotherapy and RP exhibit similar BCR rates and have significant impacts on quality of life, deciding between these two modalities is primarily driven by clinical and patient preference rather than the molecular state of the cancer (Sundaresan and Leapman, 2025). Yet stratification using validated RNA-based genomic tests such as Decipher, Prolaris and Oncotype DX Prostate outperform routine clinical risk scores in predicting metastasis and cancer-specific mortality (Ross et al., 2014; Ingrosso et al., 2022; Cooperberg et al., 2015; Moschovas et al., 2022). In a study of 146 PCa patients post RP, Decipher changed the recommended adjuvant radiotherapy decision in 30.8% of cases (Michalopoulos et al., 2014), while another study showed that Decipher could robustly identify rapid-onset 2-year BCR in an ethnically diverse cohort of 226 patients of both white and African American men (Yamoah et al., 2025). Despite this data, these genomic tests are utilised in fewer than 2% of eligible patients (Bologna et al., 2024) due to limited expertise and high patient costs (Paller et al., 2019).

Beyond selection of definitive treatment, landmark trials implementing combination therapy with ADT as a treatment for BCR have demonstrated increased durability of response. Combination with docetaxel significantly reduced the risk of death by 28% with a 13.6-month improvement in median overall survival (OS) (Sweeney et al., 2015), whereas combination with abiraterone reduced risk of death by 37%–38% (James et al., 2017). The success of these approaches underscores the need to identify superior new therapies that can be rationally combined with ADT as a treatment for BCR, which is only achievable with guidance by molecular profiling.

This paradigm mirrors the current strategy in breast cancer, where molecular stratification via immunohistochemistry and genomic panels into luminal and other subtypes guides subtype-specific therapy (Harbeck et al., 2019). As multiple, distinct genomic alterations can contribute to the development of CRPC (Figure 1B), molecular stratification in PCa could establish a similar model: not to replace AR-targeting therapies, but to identify rational combination therapies generating more robust responses.

To this end, several actionable biomarkers have been identified that warrant routine testing. Homologous recombination deficiency (HRD), commonly caused by mutations to BRCA1/2 and ATM, occurs in about 20% of mCRPC, and identifies patients likely to benefit from PARP-inhibitors that establish synthetic lethality when applied in combination with pre-existing HRD, thus selectively killing cancer cells. Accordingly, PARP-inhibitors are already an approved therapy for mCRPC. For example, combining the PARP-inhibitor, olaparib, with ARPIs improved imaging-defined progression free-survival compared to ARPIs alone (de Bono et al., 2020). In fact, pre-clinical evidence suggests that ADT impairs homologous recombination, implying a possible synergy between ADT and PARP-inhibition as a frontline therapy for high-risk PCa (Asim et al., 2017).

Loss of the tumour suppressor PTEN, an inhibitor of pI3K/AKT/mTOR signalling, is detectable in approximately 20% of PCa tumours at RP and in 50% at CRPC, driving constitutive pro-survival signalling under ADT and contributing to therapeutic resistance (Jamaspishvili et al., 2018). These patients may benefit from AKT inhibitors such as ipatasertib or capivasertib (Sweeney et al., 2021, Fizazi et al., 2026). Mutations to onco-suppressor TP53 and cell-cycle regulator RB1 are of particular concern, as their loss is strongly associated with lineage plasticity and the transcriptional reprogramming of PCa towards neuroendocrine prostate cancer (NEPC). NEPC is a highly aggressive AR-independent subtype with poor prognosis (Ku et al., 2017; Abida et al., 2019). While these markers are not directly targetable with therapy, identification of TP53/RB1-loss and AR signalling activity could identify patients with a high risk of developing NEPC, warranting closer monitoring and potentially exclusion from AR-centric treatment strategies. Finally, 10%–15% of patients exhibit primary resistance to ADT, where AR amplifications and splice variants such as AR-V7 predict treatment resistance (Varenhorst et al., 2016; Antonarakis et al., 2014). These patients have poorer outcomes, and early identification of AR status could identify these non-responsive patients for other treatments, such as taxane-based therapy. Comprehensive molecular profiling of diagnostic biopsy tissue for actionable alterations via genomic panels or immunohistochemistry thus has high potential to identify rational treatment combinations with ADT or salvage radiotherapy to address BCR in PCa. However, as primary PCa is typically comprised of multiple, genetically independent foci (Carm et al., 2019), this initial heterogeneity will make stratification significantly harder, warranting sampling and molecular testing of all available tissue. Mimicking the evolution of breast cancer treatment, this would progress PCa management from a ‘one size fits all’ approach towards true personalised therapy in order to improve patient outcomes despite their diverse disease (molecular) journeys.

## CRPC mechanisms of resistance

Although initially efficacious in terms of improving survival–response rates are approximately 90% for ADT in CSPC, and still 50%–60% for enzalutamide in CRPC (Scher et al., 2012) – blocking AR signalling provides a strong selective pressure that drives resistance through both intrinsic and acquired mechanisms (Watson et al., 2015).

The majority of CRPC remains driven by AR signalling, even under low androgen levels or direct inhibition. AR gene amplification occurs in 40%–60% of CRPC, with amplification of the AR-enhancer region in 81% of mCRPC patients post ADT (Quigley et al., 2018; Robinson et al., 2015) (Figure 1B). Significantly, this amplification of AR is predominantly detected post-ADT, indicating that it is a response to the selective pressure of hormone therapy (Le et al., 2023). Similarly, AR mutations increase from 0%–4% of localised PCa to 10%–20% in mCRPC (Wyatt and Gleave, 2015), primarily affecting the ligand-binding domain, leaving AR constitutively active, or enabling activation by glucocorticoids or even by ARPIs (Sumiyoshi et al., 2021). AR splicing variants, most notably AR-V7, lack the ligand binding domain and can be constitutionally active. Again, these variants arise through aberrant RNA splicing and are selected for as a consequence of ADT (Henzler et al., 2016).

As noted above, resistance can also occur through lineage plasticity away from luminal AR-driven cancer towards NEPC which bypasses AR signalling entirely. Emerging evidence suggests ARPIs promote this trans-differentiation, since AR maintains luminal epithelial identity through extracellular matrix/integrin/YAP/TEAD signalling. Thus, suppression of AR inhibits this, facilitating a switch towards transcription factor ASCL-driven neuroendocrine programs, particularly in cells with TP53/RB1 loss (Han et al., 2025).

CRPC also exhibits profound intra- and inter-tumour heterogeneity, known to be the main cause of treatment failure (Fu et al., 2025). A single tumour has spatially and temporally distinct subclones that harbour divergent resistance mechanisms that evolve over time, fundamentally complicating therapeutic targeting (Gundem et al., 2015). CRPC is typically immunologically ‘cold’, with sparse CD8^+^ (Cytotoxic) T cell infiltration, low neoantigen load and presentation, as well as enriched immunosuppressive myeloid populations. In the absence of personalised tumour and molecular profiling, these factors collectively underpin poor response rates to immune checkpoint inhibitors for the overall CRPC patient population (Kwon and Joung, 2025). Yet within this broad CRPC patient population, 3%–5% of tumours exhibit high tumour mutational burden (TMB) or microsatellite instability; features that are associated with strong immunotherapy responses (Abida et al., 2019, Antonarakis et al., 2019). Similarly, several well-established oncogenic pathways such as EGFR/MAPK, PI3K/AKT/mTOR, JAK/STAT, WNT/β-catenin and MYC signalling can drive CRPC by replacing AR signalling (Traish and Morgentaler, 2009; Shorning et al., 2020; Deng et al., 2022; Murillo-Garzón and Kypta, 2017; Qiu et al., 2022). These pathways can confer resistance in distinct cell sub-populations, even within a single tumour. As with AR-targeted therapies, resistance arises to therapies targeting these pathways as well. PARP inhibitor response can become limited by *BRCA 1/2* reversion mutations that restore HR, as well as replication fork stabilisation (Cheng et al., 2018; Lin et al., 2019). More broadly, targeted therapies fail through either ‘on-target’ kinase mutations or ‘off-target’ activation of alternate pathways, as characterised for EGFR and ALK-targeted agents (Cooper et al., 2022).

This heterogeneity reinforces the fact that CRPC is not a single disease but rather a constellation of co-evolving molecular states that require comprehensive profiling to adequately characterise and treat. While individually none of these therapies serve as a ‘silver-bullet’ for CRPC, if paired with sufficient \ longitudinal monitoring, these evolutionary ‘moves’ arising from CRPC adaptation can be met with molecularly-guided ‘counter-moves’ for more effective personalised treatment, whereby clinicians match the tumour’s current molecular state to the best treatment (Abida et al., 2019) (Figure 1).

## Current therapies and monitoring for mCRPC

### Therapies

Current mCRPC treatment options include the next-generation taxane, cabazitaxel, specifically effective for patients who have developed efflux pump-driven docetaxel resistance. Cabazitaxel showed improved clinical outcomes compared to a second ARPI (de Wit et al., 2019), however its use is limited as 81.7% of patients experience grade ≥3 neutropenia (de Bono et al., 2010). For the >70% of CRPC patients that express PSMA as detected via PSMA-PET, the theranostic radionucleotide Lutetium-177 (^177^Lu)-PSMA-617 is a new mCRPC therapy that binds selectively to prostate cells and delivers a beta-radiation payload to cause DNA damage (Nindra et al., 2024), resulting in prolonged image-based progression free survival (PFS) and OS (Sartor et al., 2021). For patients that have undergone genetic testing and are identified as having *BRCA1/2* or *ATM* alterations (or a similar HR deficiency), PARP-inhibitors also improved OS beyond ARPIs-alone (Hussain et al., 2020). However, none of these therapies are curative, only extending OS by a matter of months.

For AR-driven CRPC, promising new therapies include degradation of AR through proteolysis-targeting chimeras (PROTACs), which consist of an AR-binding warhead linked to an E3 ubiquitin ligase that targets AR for ubiquitination and subsequent degradation. Currently, the most promising of these is ARV-766, which targets wild-type AR as well as common mutations to the ligand-binding domain (LBD); present in 20%–25% of mCRPC patients post ARPI treatment. In an ongoing phase I/II clinical trial, 50% of biomarker-selected patients harbouring AR ligand-binding domain mutations achieved a 50% decline in PSA (PSA50) (Petrylak et al., 2024). Addressing the AR axis from a different perspective, preclinical investigations suggest that targeting the ASCL1-driven NEPC transcriptional program through TEAD-inhibition may redirect cell differentiation toward a luminal prostate lineage, restoring sensitivity to standard AR-targeted therapies (Romero et al., 2024; Han et al., 2025). In addition, inhibition of novel pathways, such as the interaction between secreted phospholipase A_2_ IIA (hGIIA) and both EGFR and vimentin, can supress CRPC tumour growth in preclinical models. An inhibitor of this interaction is currently in a Phase I/II clinical trial (Mann et al., 2025). These established and emerging therapeutics constitute an increasingly diverse arsenal of tools with which to counteract the adaptability of CRPC. Yet realising their full potential demands that the right treatment reaches the right patient at the right time.

### Imaging

In addition to monitoring PSA, PSMA-PET scans are routinely used for restaging after BCR, offering superior sensitivity and specificity over conventional imaging (Hofman et al., 2020). However, PSMA expression is also reduced in NEPC and low tumour volume PCa thereby yielding far lower rates of detection in low PSA (42% when PSA <0.2 ng/mL) compared to higher PSA PCa (97% when PSA > 2 ng/mL) (Perera et al., 2016). This low PSA/PSMA phenotype is problematic to track, but testing can be supplemented by Fluorodeooxyglucose (FDG)-PET scans, which detect metabolically active cancers. Indeed, dual PSMA and FDG scanning highlighting 18.8% of CRPC patients as having PSMA-negative but FDG-positive lesions (Pan et al., 2024).

### Liquid biopsy for improved detection, profiling and personalised treatment of CRPC

Maximising patient outcomes by matching them to the right treatment requires longitudinal molecular monitoring that can safely and repeatedly track tumor vulnerabilities as they evolve, ideally at the single-cell level. Although tumour tissue remains the gold-standard substrate for molecular profiling, solid biopsies are invasive and thus not compatible with the longitudinal monitoring necessary for precision targeting of the ever-evolving tumor. Exemplifying this, molecular profiling of archival tissue biopsies obtained before AR inhibition, sometimes many years prior to a current treatment decision, is unlikely to reliably encapsulate the molecular state of a cancer given the likelihood of extensive adaptation under the selective pressure of treatment. Thus, in addition to PSA-measurement and PSMA-PET-imaging to track disease burden, molecular profiling via longitudinally compatible liquid biopsy–particularly via blood-based sampling–is emerging as a potentially invaluable complement to initial solid tissue biopsy; enabling molecular and/or phenotypic tracking of CRPC over the diverse and dynamic disease journey of each patient.

### Improving response monitoring beyond PSA

Blood-analyte PSA alone is insufficient for monitoring response in CRPC, since approximately 30% of CRPC tumors no longer rely on the AR signalling that induces PSA-production, resulting in false negative readings. Supplementary serum markers such as chromogranin A or neuron-specific enolase (NSE) can supplement PSA monitoring by identifying neuroendocrine differentiation in advanced PCa (Heck et al., 2017), while carcinoembryonic antigen (CEA) is elevated in aggressive, low PSA PCa, correlating with poor prognosis (Bray et al., 2022). Concurrent measurement of these markers can be conducted from the same blood sample, improving clinical confidence in assessing CRPC treatment response.

### Circulating tumour DNA

Fragments of tumour-derived DNA circulating in the blood, known as circulating tumour DNA (ctDNA), provide another avenue for molecular monitoring of CRPC mediated by liquid biopsy (Wan et al., 2017). Unlike PSA or PSMA, ctDNA is not dependent on AR signalling or preservation of prostate adenocarcinoma lineage, presenting as a robust avenue for tracking the progression of NEPC or other low PSA/PSMA mCRPC (Annala et al., 2021) given close correlation between detection rates and metastatic burden (Conteduca et al., 2025). ctDNA is detectable in 94% of patients with advanced disease (Tukachinsky et al., 2021), and technologies now exist to distinguish primary from metastatic disease with 98.9% accuracy, as well as to detect ctDNA fractions as low as 2% of total cell-free DNA (Chen et al., 2022).

Beyond detection, ctDNA from minimally invasive blood samples can be used for longitudinal monitoring of CPRC progression, providing real time molecular characterisation of the cancer phenotypic state, such as NEPC status, or of targetable and clinically relevant genetic mutations. ctDNA detection of HR alterations such as *BRCA1/2* and *ATM* is now an FDA approved method to predict response to PARP inhibitors (Mateo et al., 2020), whereas detection of *AR, TP53, RB1* or *PTEN* alterations or NEPC status are prognostically informative but not yet routinely used to direct therapy.

### Circulating tumour cells

Analysis of circulating tumour cells (CTCs) is a complementary liquid biopsy strategy, offering single-cell-resolved information not delineated by ctDNA, thus encapsulating the heterogeneity that is a key mechanism of CRPC’s resistance to treatment (Mizuno et al., 2025). CTCs are the ‘seeds of metastasis’, acting as a functional metastatic biomarker such that their enumeration in blood is an established FDA approved clinical practice used to stage patients with advanced and metastatic disease, correlating with disease burden and outcomes (Liu et al., 2017). Interestingly, the CRPC associated AR-V7 transcript is an emerging biomarker, more effectively identified from CTCs in comparison to circulating or exosomal AR-V7 mRNA (Nimir et al., 2019), and its detection in liquid biopsy is strongly associated with AR-targeted therapy outcome (Khan et al., 2022). Beyond DNA or RNA analysis in CTCs, monitoring of proteins can provide a more direct and functional measure of oncogenic signalling, with key protein markers such as nuclear localisation of AR-V7 (Scher et al., 2017) and PSMA (Gorges et al., 2016), representing promising biomarkers that predict response in clinical trials but are not yet widely implemented in the clinic.

Characterization of CTCs has long held great potential as a guide for precision medicine–allowing a direct readout of the signalling pathways driving CRPC in each cell, in each patient, at each timepoint. Yet in reality, CTC utilisation has been hampered primarily by two key factors–their rarity and the low-plexity (number of molecular markers concurrently measured per cell) of biomarker measured per cell, especially in relation to proteins (Pantel and Alix-Panabières, 2025).

CTCs are indeed rare, with just 5 CTCs per 7.5 mL of blood used as a threshold for clinically unfavourable PCa (Thalgott et al., 2013). CTC enrichment strategies fall into four approaches: positive selection using biomarkers such as EpCAM, e.g., CellSearch, negative selection via depletion of non-tumour blood cells that reduces exclusion bias, size-based physical separation, which is independent of biomarker expression, or a ‘no cell left behind’ approach, where all nucleated cells from whole blood are analysed. To increase CTC retrieval further, leukapheresis might be the ultimate strategy, whereby this standard clinical procedure could be used to directly isolate nucleated cells that include thousands of CTCs, while leaving plasma and RBCs untouched (Stoecklein, 2023; Dai et al., 2025). Initial tests assessing 1–2 L of blood achieved CTC recovery with a mean of 10,057 CTCs (range 100–58,125) (Mishra et al., 2025). While still investigational and not routinely implemented and requiring adequate peripheral venous access, this strategy could be implemented when the onset of CRPC is detected–particularly before administration of taxanes or radioligands that can cause complications during larger blood draws–enabling detailed CTC analyses that can guide personalised therapy-selection.

Analysis of key protein expression by CTCs is most commonly achieved via immunofluorescence (IF) imaging. Yet the low-plexity of standard IF restricts molecular marker monitoring to a gold-standard of just 4–6 markers (Thiele et al., 2017), with a maximum reported of 15 markers (Whalen et al., 2024). Mass cytometry has recently been applied to increase marker plexity to ∼20 proteins-of-interest per cell (Bose et al., 2025), and while a powerful advance, this technique does not resolve the sub-cellular localization of proteins (e.g. inactive cytoplasmic AR *versus* active nuclear AR), thus missing a critical dimension of functional status. Other high-plex CTC assays include the 15-plex *in situ* RNA detection (Bonstingl et al., 2024), which captures transcriptional heterogeneity, but being transcript-based, is an indirect measurement of protein level expression and pathway activation. Our group has recently presented a prospective pipeline achieving multiplexed immunofluorescence profiling of CTCs, capturing the expression and sub-cellular localisation of 50 (phospho)protein biomarkers per cell. This yielded quantitative signatures of therapy non-response as well as identifying therapeutically actionable biomarkers. This approach requires clinical validation, however, provides a proof-of-principle for using CTCs to characterise tumour heterogeneity and potentially guide rational selection of combination therapies (Mann et al., 2025). While liquid biopsy-based ctDNA and CTC analyses constitute promising avenues towards contemporaneous molecular profiling of CRPC, both can be constrained by low shedding in some cancers, as well as platform variability. Further analytical optimisation (reproducibility, cross-platform standardisation, input noise assessment) and clinical validation of biomarkers on a per-treatment basis are required before the higher costs of testing are sufficiently justified to motivate routine clinical implementation.

### Molecular profiling for rational selection of targeted therapies to treat CRPC

**FIGURE 2:**
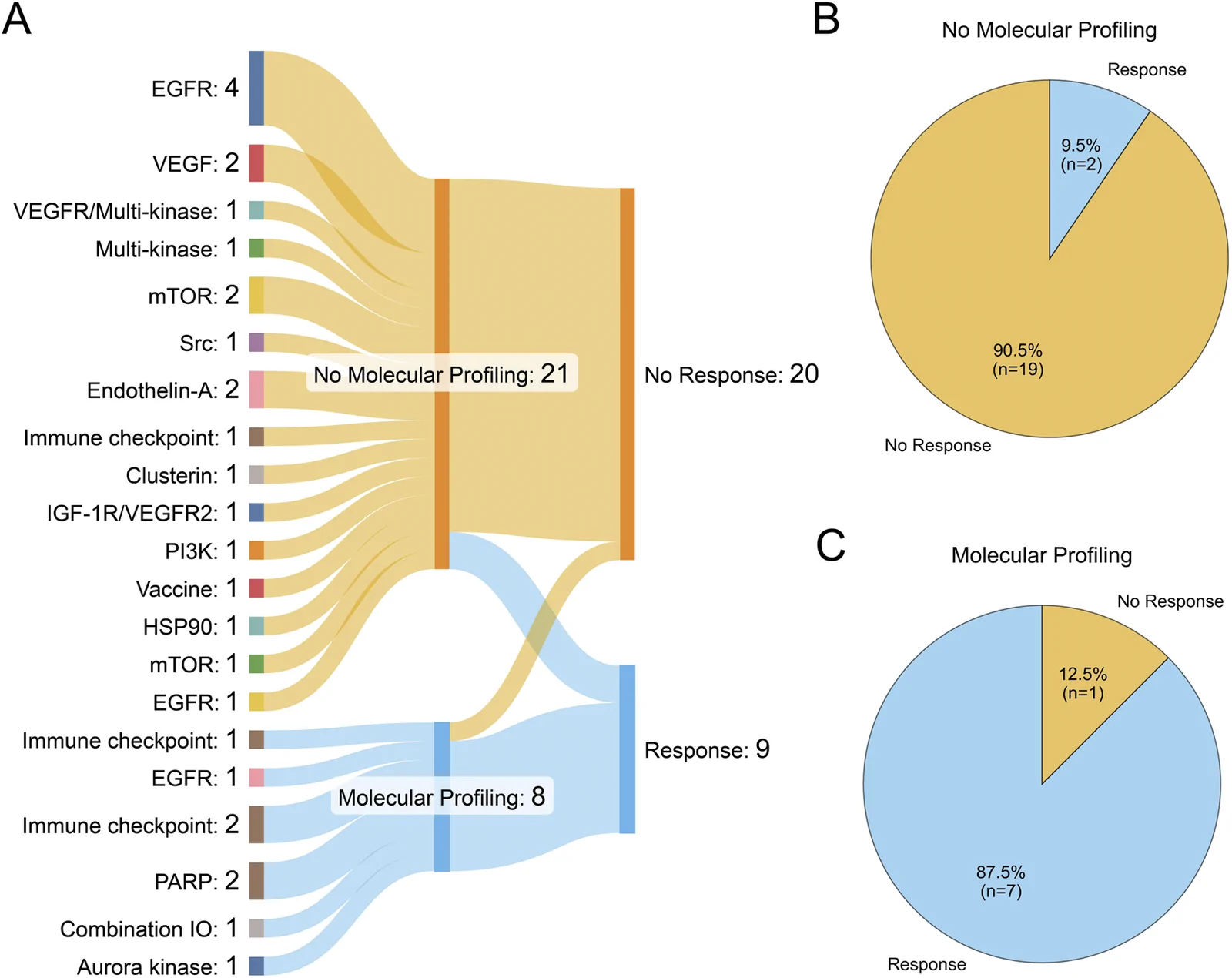
Phase II/III clinical trials of targeted therapies in CRPC, highlighting correlation between molecular profiling of patient cohort and identification of response. **(A)** Sankey plot of targets (left) for identified clinical trials in CRPC, showcasing which underwent molecular profiling (middle) and response (right). Pie charts show response rates of clinical trials with **(B)** no molecular profiling and **(C)** molecular profiling, respectively.

With CRPC still classified as incurable, it is critical that precision diagnostics are implemented in order to help deploy and direct the arsenal of targeted therapies being successfully applied in other cancers. Many of these targeted therapies, such as those inhibiting EGFR, VEGF/VEGFR, Src, mTOR, immune checkpoints, PI3K, HSP90, Aurora kinase, IGF-1R, Endothelin-A and therapeutic vaccines, have already been trialled for CRPC treatment, as summarised in Figure 2A and detailed in Supplementary Table 1. Unfortunately, the majority of these trials have failed to demonstrate efficacy, being unable to improve patient outcomes beyond standard-of-care ARPIs (alone or in combination). Importantly, however, *the vast majority of these clinical trials did not stratify patients based on measurement of biomarkers indicative of improved likelihood of response to treatment* (Figure 2B). This is crucial to note since most of these therapies are expected to benefit only a subset of patients (as per Figure 1B), such that positive effects within applicable patient subsets could easily be masked in a general CRPC population. Thus, the failure of these trials highlights a deficit in contemporaneous molecular profiling that prevents appropriate patient stratification, leaving doubt as to the reported insufficiency of the therapies themselves.

Notably, the majority of trials that conducted molecular profiling for targeted stratification, either by prospective patient stratification or post-trial analysis of response, showed positive outcomes (Figure 2C). For example, the EGFR inhibitor cetuximab was salvaged only significantly improved PFS in a subset of patients with profiled EGFR overexpression and persistent PTEN expression; while most other clinical trials of unselected populations failed with EGFR inhibitors (Cathomas et al., 2012; Figure 2). The aurora kinase inhibitor alisertib was administered as a monotherapy to patients with NEPC, where a subset of patients with high N-MYC and Aurora-A overactivity exhibited complete response, including resolution of liver metastases (Beltran et al., 2019). Similarly, trials for the immunotherapy PD-L1 inhibitor pembrolizumab saw significantly increased survival in high PD-L1 versus low PD-L1 expressing cohorts (Antonarakis et al., 2020). This was also seen in a combination therapy of PD-L1 inhibitor nivolumab with CTLA-4 inhibitor ipilimumab, with objective response rate of high PD-L1 cohort at 25% and the low PD-L1 cohort at 10% (Sharma et al., 2020). Selection of patients for the PARP inhibitor niraparib in combination with the ARPI abiraterone based on HR deficiencies was also effective, with patients with HR gene alterations having significantly longer PFS (16.6 months) compared to those without HR alterations (10.9 months) (Chi et al., 2023). This culminated in a phase III clinical trial of niraparib with abiraterone for patients with mCRPC harbouring BRCA1/2 mutations, which also significantly improved survival (Attard et al., 2025). Furthermore, the high intratumor heterogeneity and plasticity of CRPC suggests that single agent therapies are unlikely to result in durable control, further highlighting the need for molecular profiling to select rational combination therapies, as well as longitudinal tracking to adjust treatment in response to continuous tumour adaptation.

## Outlook

Together, these findings and outcomes make a compelling case for dramatically improved and expanded molecular profiling of CRPC. This may optimise treatment of patients using the spectrum of already approved CRPC therapies, while also enabling better stratification of patients to suitably targeted therapy trials. Of particular importance, advances in profiling of liquid biopsy (e.g. blood)-derived analytes such as ctDNA and CTCs offers avenues to guide targeted therapy selection without reliance on repeat (invasive and possibly inaccessible for metastatic bone disease) biopsies or on archival tissue samples where profiling may no longer accurately delineate current therapeutic vulnerabilities. Instead, minimally invasive liquid biopsies can facilitate safe longitudinal molecular monitoring at almost any stage of the disease journey. Precision oncology–utilizing longitudinal liquid biopsy based molecular profiling for the rational selection of therapies and, in particular, combination therapies–represents a particularly promising strategy for the treatment of heterogeneous and phenotypically plastic CRPC.

## Glossary

177Lu: Lutetium-177
ADT: Androgen Deprivation Therapy
AR: Androgen Receptor
ARPI: Androgen Receptor Pathway Inhibitor
AR-V7: Androgen Receptor Splice Variant 7
BCR: Biochemical Recurrence
CEA: Carcinoembryonic Antigen
cfDNA: Cell-Free DNA
CRPC: Castration-Resistant Prostate Cancer
CSPC: Castration-Sensitive Prostate Cancer
CTC: Circulating Tumour Cell
ctDNA: Circulating Tumour DNA
DDR: DNA Damage Response
DHT: Dihydrotestosterone
FDG-PET: Fluorodeoxyglucose Positron Emission Tomography
HRD: Homologous Recombination Deficiency
IO: Immuno-Oncology
LBD: Ligand Binding Domain
LHRH: Luteinising Hormone-Releasing Hormone
mCRPC: Metastatic Castration-Resistant Prostate Cancer
NEPC: Neuroendocrine Prostate Cancer
NSE: Neuron-Specific Enolase
OR: Objective Response
OS: Overall Survival
PARP: Poly(ADP-Ribose) Polymerase
PCa: Prostate Cancer
PET: Positron Emission Tomography
PFS: Progression-Free Survival
PI-RADS: Prostate Imaging Reporting and Data System
PROTAC: PROteolysis TArgeting Chimera
PSA: Prostate-Specific Antigen
PSA50: ≥50% Decline in Prostate-Specific Antigen
PSMA: Prostate-Specific Membrane Antigen
RNA-seq: RNA Sequencing
rPFS: Radiographic Progression-Free Survival
RP: Radical Prostatectomy
SEER: Surveillance, Epidemiology, and End Results Program
SOC: Standard of Care
TMB: Tumour Mutational Burden
TNM: Tumour–Node–Metastasis Classification
