# Supplementary material for: The role of molecular profiling for castration-resistant prostate cancer treatment and therapy development
Source: Front Cell Dev Biol. 2026 Aug 21;14:1894354. doi: 10.3389/fcell.2026.1894354 (PMC13542128; doi:10.3389/fcell.2026.1894354)
Supplement: Supplementary file 1 [file Table1.docx]

**Supplementary Table 1: Phase II/III clinical trials of targeted therapies in CRPC. Colour indicates whether molecular testing or stratification was conducted (green = yes, red = no) and whether the clinical trial identified a responding population (green = yes, red = no).**

| **Target** | **Agent(s)** | **Phase** | **Treatment** | **Molecular Testing/Stratification** | **Endpoints** | **Outcome** | **Safety Concerns** | **Ref** |
| --- | --- | --- | --- | --- | --- | --- | --- | --- |
| EGFR | Gefitinib | II | Monotherapy | No molecular selection; EGFR expression not required | PSA | No objective responses; median PFS 2.0 months; did not meet efficacy endpoint | Generally well-tolerated; grade 3-4 toxicity 15% | Small et al., 2007 |
| EGFR | Gefitinib | II | Monotherapy | No EGFR mutation testing performed | PSA | Minimal activity as monotherapy; <5% response rate | Rash, diarrhea typical of EGFR-TKIs | Canil et al., 2005 |
| EGFR | Gefitinib | II | Monotherapy | None | PSA | 2% Response rate | Infusion reactions, skin toxicity | Pezaro et al., 2009 |
| EGFR | Cetuximab | II | Combination with docetaxel | Evaluation of EGFR, PTEN amphiregulin, epiregulin was conducted | PSA | Possible improvement in patient outcome and potential correlation with response and EGFR overexpression +persistent PTEN expression. | 47% of patients experienced grade 3 toxicities, 8% grade 4 | Cathomas et al., 2012 |
| EGFR | Erlotinib | II | Combination with Docetaxel | None | OS/PSA | No observable benefit over docetaxel monotherapy. | Some Hematologic toxicity | Gross et al., 2007 |
| EGFR | Erlotinib | II | Monotherapy | None | PSA/Clinical progression | Clinical benefit in 40% of patients | Diarrhea and cutaneous toxicity | Gravis et al., 2008 |
| VEGF | Bevacizumab + docetaxel | III | Combination with SOC (docetaxel + prednisone) | No biomarker stratification | OS/PFS/PSA/OR | Improved PFS (9.9 vs 7.5 months) but NO OS benefit; primary endpoint not met | Increased hypertension, bleeding, proteinuria | Kelly et al., 2012 |
| VEGF | Aflibercept + docetaxel | III | Combination with SOC (docetaxel + prednisone) | No biomarker selection | OS | No improvement in OS (22.1 vs 21.8 months); increased toxicity | Significant increase in grade 3-5 adverse events (64% vs 50%) | Tannock et al, 2013 |
| VEGFR/Multi-kinase | Sunitinib | III | Combination with SOC or maintenance monotherapy | No molecular stratification | OS/PFS | Failed to improve OS in combination or maintenance settings | Fatigue, hand-foot syndrome, cytopenias | Michaelson et al., 2014 |
| Multi-kinase | Sorafenib | II/III | Monotherapy or combination | No predictive biomarkers used | PSA/Radiographic | No significant benefit as monotherapy or in combination, although discordant response measurements. | Hand-foot skin reaction, diarrhea, hypertension | Dahut et al., 2008 |
| mTOR | Everolimus | II | Monotherapy | Pathologic evaluation identified inhibition of downstream ps6 post treatment, but continued Ki67. | PSA | Minimal single-agent activity despite downstream mTOR target inhibition. | Mucositis, fatigue anorexia. | George et al., 2020 |
| mTOR | Everolimus | II | Combination with bicalutamide | None | PSA | 2.5 months longer median duration of PSA response with combo vs bicalutamide alone | 54%of patients experienced grade 3-4 toxicities. | Chow et al., 2016 |
| mTOR | Temsirolimus | II | Monotherapy | >5 CTCs, No molecular selection for PTEN loss or PI3K pathway activation | PSA/CTCs | No improvement in PFS or OS | Metabolic toxicity, mucositis | Armstrong et al., 2013 |
| Src | Dasatinib | III | Combination with SOC (docetaxel + prednisone) | No molecular biomarkers; Src activity not assessed | OS | No improvement in OS (21.5 vs 21.2 months) | Diarrhea | Araujo et al., 2013 |
| Endothelin-A | Atrasentan | III | Combination with docetaxel | No molecular stratification | OS/PFS | No significant OS benefit; early trial terminated | No significant increase in toxicity | Quinn et al., 2013 |
| Endothelin-A | Zibotentan | III | Combination with docetaxel | No biomarker selection | OS/PSA/PFS | No OS benefit in combination with docetaxel | Fluid retention, peripheral edema | Fizazi et al., 2013 |
| Immune checkpoint (CTLA-4) | Ipilimumab | III | Monotherapy (after bone-directed radiation) | No biomarker stratification; TMB/MSI not assessed | OS | Improved PFS but NO OS benefit vs placebo after radiation | Immune-related adverse events including colitis, hepatitis | Kwon et al., 2014 |
| Immune checkpoint (PD-1) | Pembrolizumab | II | Monotherapy | 3 cohorts with positive, mixed and negative PD-L1 expression. Exploratory whole exome sequencing of tumors. 19% had aberrations to HDR. | OS | PD-L1 positive OS 9.5 months, PD-L1 negative cohort OS 7.9 months, Bone-predominant disease (mixed PD-L1) 14.1 months | Acceptable - Immune-related adverse events | Antonarakis et al., 2020 |
| Immune checkpoint (PD-1) | Nivolumab | II | Monotherapy | DDR assessed using ctDNA/whole exome sequencing, deficiencies identifiable in 30.5% | PSA/PFS/OS | Clinical activity in a subset of patients, however DDR deficiencies did not predict response. | 47.3% had grade 3 adverse events. | Isaacsson Velho et al., 2024 |
| Immune checkpoint (PD-L1) | Atezolizumab + enzalutamide | III | Combination with enzalutamide | PD-L1 expression, HRD status, PTEN status assessed in archival tissue. | OS | No improvement in OS vs enzalutamide alone in unselected 1L mCRPC. Longer PFS seen in high PD-L1 IC2/3, CD8 expressing, linking immune genes such as CXCL9 and TAP1 | Acceptable increased immune-related toxicities | Powles et al., 2022 |
| Clusterin | Custirsen | III | Combination with docetaxel + prednisone | No molecular stratification | OS | No improvement in OS for custirsen 23.4 months vs doxetaxel alone 22 months. | Neutropenia | Chi et al., 2017 |
| IGF-1R and VEGFR2 | Cixutumumab and ramucirumab | II | Combination of either Cixutumumab or ramucirumab with docetaxel | No molecular selection | cPFS/OS/ | cPFS for cixutumumab 4.1 months, for ramucirumab 6.7 months. | Fatigue | Hussain et al., 2015 |
| PI3K | Buparlisib + enzalutamide | II | Monothereapy and combination with enzalutamide | No molecular selection | PFS | No significant activity. | weight loss, diarrhea, nausea | Armstrong et al., 2017 |
| PARP (unselected) | Niraparib | III | Combination with abiraterone + prednisone | HRD status assessed by tissue/blood; negative cohort showed no benefit | rPFS/OS | Significantly improved PFS in mCSPC patients with BRCA1/2 mutations and other HR deficiencies. | Generally manageable but anemia, thrombocytopenia | Attard et al., 2025 |
| PARP | Niraparib | III | Combination with abiraterone + prednisone | DDR assessed by tissue/plasma arrays into 2 cohorts - either positive or negative for HRD alterations | rPFS | Median rPFS in HRD+ group 16.6 months signficiantly higher than without Niraparib 10.9 months. | Tolerable anemia and hypertension | Chi et al., 2023 |
| Vaccine (PROSTVAC) | PROSTVAC-VF | III | Monotherapy (active immunotherapy) | No immune biomarkers predictive | OS | No improvement in OS (34.4 vs 34.3 months vs physician's choice) | Generally well-tolerated; injection site reactions | Gulley et al., 2019 |
| Combination IO | Ipilimumab + nivolumab | II | Combination immunotherapy (no SOC) | whole exome sequencing. | OS | ~25% response rate with 4% showing complete response. Improved response in patients with high tumor mutational burden. In patients with HRD, overall response rate was 50% vs 22% | immune-related adverse events (grade 3-4 in >50%) | Sharma et al., 2020 |
| HSP90 | Retaspimycin (IPI-504) | II | Monotherapy | No molecular selection | PSA | Trial terminated due to toxicity | unaxxeptable toxicity. 2 drug related deaths. | Oh et al., 2011 |
| Aurora kinase | Alisertib | II | Monotherapy | Eligibility required NEPC phenotype. Archival biopsies underwent whole exome and RNA-seq. Patient derived organoids were developed. | rPFS | Six month rPFS was 13.4%, four patients with N-myc and Aurora-A overactivity responded exceptionally (resolution of liver metastases and prolonged stable disease). Organoids exhibited concordant responses. | Neutropenia, stomatitis, fatigue | Beltran et al., 2019 |
